# Role of TRPM8 in dorsal root ganglion in nerve injury-induced chronic pain

**DOI:** 10.1186/1471-2202-12-120

**Published:** 2011-11-23

**Authors:** Lin Su, Chao Wang, Yong-hao Yu, Yong-ying Ren, Ke-liang Xie, Guo-lin Wang

**Affiliations:** 1 Department of Anesthesiology, General Hospital of Tianjin Medical University, Anshan Road No. 154, Heping District, Tianjin, 300052, China

## Abstract

**Background:**

Chronic neuropathic pain is an intractable pain with few effective treatments. Moderate cold stimulation can relieve pain, and this may be a novel train of thought for exploring new methods of analgesia. Transient receptor potential melastatin 8 (TRPM8) ion channel has been proposed to be an important molecular sensor for cold. Here we investigate the role of TRPM8 in the mechanism of chronic neuropathic pain using a rat model of chronic constriction injury (CCI) to the sciatic nerve.

**Results:**

Mechanical allodynia, cold and thermal hyperalgesia of CCI rats began on the 4th day following surgery and maintained at the peak during the period from the 10th to 14th day after operation. The level of TRPM8 protein in L5 dorsal root ganglion (DRG) ipsilateral to nerve injury was significantly increased on the 4th day after CCI, and reached the peak on the 10th day, and remained elevated on the 14th day following CCI. This time course of the alteration of TRPM8 expression was consistent with that of CCI-induced hyperalgesic response of the operated hind paw. Besides, activation of cold receptor TRPM8 of CCI rats by intrathecal application of menthol resulted in the inhibition of mechanical allodynia and thermal hyperalgesia and the enhancement of cold hyperalgesia. In contrast, downregulation of TRPM8 protein in ipsilateral L5 DRG of CCI rats by intrathecal TRPM8 antisense oligonucleotide attenuated cold hyperalgesia, but it had no effect on CCI-induced mechanical allodynia and thermal hyperalgesia.

**Conclusions:**

TRPM8 may play different roles in mechanical allodynia, cold and thermal hyperalgesia that develop after nerve injury, and it is a very promising research direction for the development of new therapies for chronic neuroapthic pain.

## Background

Chronic neuropathic pain is a refractory pain characterized by its complex mechanisms and diverse clinical manifestations [1]. Traditional therapies usually bring about many side effects [2]. Moderate cold stimuli can relieve pain [3], which provides an inspiration for developing new treatments of chronic pain. Recently, transient receptor potential (TRP) channel family has been proposed to play an important role in thermosensation in mammals. Six thermosensitive ion channels of this family have been discovered, including TRPV1, TRPV2, TRPV3, TRPV4, TRPM8 and TRPA1. Among them, TRPM8 and TRPA1 are responsive to cold stimuli [4]. TRPM8 is a ligand-gated non-selective cation channel involved in detection of sensations such as coolness. It is permeable to monovalent cations sodium, potassium, and cesium and divalent cation calcium. TRPM8 is activated by cooling and exogenous chemicals such as menthol and icilin, with an activation temperature of approximate 25-28°C [5-9]. In contrast, TRPA1 is activated at about 17°C [10,11]. This channel is not required for the transduction of cold sensation in physiological conditions [12,13], but it is involved in mediating cold hypersensitivity after inflammatory injury [14]. All these findings greatly promote the progress in exploring the relationship between temperature and pain. Studies have shown that levels of TRPM8 protein [15] and RNA [16] were both increased in rats with chronic neuropathic pain. In addition, inflammatory factors such as H^+^, bradykinin and phospholipase [17-19], and altering intracellular pH [17] can both affect the activation of TRPM8. These suggested that TRPM8 ion channel may be closely related to hyperalgesia induced by neurological diseases and inflammation.

In previous studies, much attention has been focused on the role of TRPM8 in the formation of cold hyperalgesia caused by nerve injury. For example, TRPM8 was proposed to induce increased sensitivity to cold in mice with chronic neuropathic pain [20]. However, some investigators suspected this point of view and put forward that compared with TRPM8, TRPA1 was more likely to play a substantial role in the mechanism of chronic nerve injury-induced cold hyperalgesia [21]. In contrast, another study suggested that neither TRPM8 nor TRPA1 was likely to contribute directly to cold hyperalgesia in rats with nerve injury [22]. So far, there are few studies exploring whether or not TRPM8 plays a similar role in mechanical allodynia, cold and thermal hyperalgesia, which are most commonly seen clinically that develop after nerve injury. In this study, we simultaneously test the alteration of cold, mechanical and thermal sensitivity in chronic constriction injury (CCI) model of neuropathic pain in rats, which facilitates the comparative study of the role of TRPM8 in the mechanism of these three different types of sensitized pain responses.

In the present study, we firstly demonstrate the variation trend of TRPM8 protein expression in L5 dorsal root ganglia (DRG) ipsilateral to nerve injury with the development and maintenance of pain hypersensitivity of the operated hindpaw of CCI rats. Thereafter how activation or inhibition of TRPM8 channel influences the behavioral sensitization of CCI rats and the expression of TRPM8 protein in DRG is investigated.

## Results

### Alteration of TRPM8 protein expression following CCI

This part of the experiment is used to investigate whether the alteration in TRPM8 level is related to the mechanism of chronic neuropathic pain. We firstly explore the behavioral performance of hyperalgesia after CCI. Cold and thermal hyperalgesia and mechanical allodynia were measured before operation (baseline) and on the 1st, 4th, 7th, 10th and 14th day after operation.

**Figure 1:**
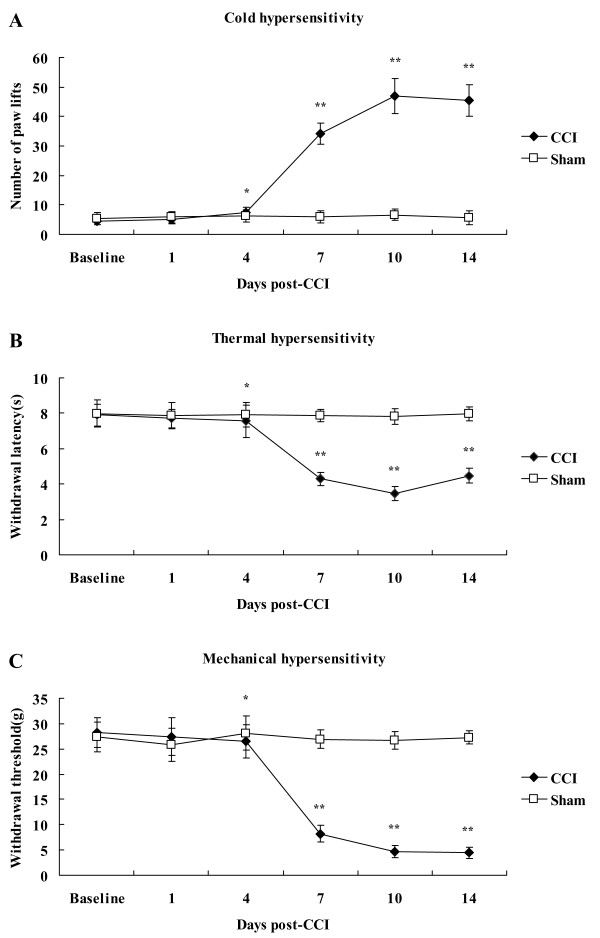
**Assessment of behavioral reflex sensitization in CCI model of neuropathic pain**. A, Cold sensitivity assessed by cold plate test. B, Thermal sensitivity assessed by hot plate test. C, Mechanical sensitivity assessed by Von Frey filament assay. Data are expressed as mean ± SEM. *P < 0.05, **P < 0.01 vs. sham-operated rats (t test).

Cold hyperalgesia was measured using the cold plate test. Compared with the total number of brisk lifts of operated hind paw in sham-operated rats, the value for brisk lifts of CCI rats was significantly increased on day 4 postligature placement (7.33 ± 2.01 vs 6.16 ± 2.02, *P *< 0.05) (Figure 1A). This difference reflected a heightened sensitivity to cold stimuli. The increased number of brisk lifts persisted through day 7 (34.17 ± 3.68 vs 6.00 ± 2.10, *P *< 0.01), and reached the peak on day 10 (46.83 ± 5.98 vs 6.66 ± 1.99, *P *< 0.01), and remained increased on day 14 following ligation (45.33 ± 5.36 vs 5.66 ± 2.36, *P *< 0.01). Thermal hyperalgesia was assessed by hot plate test. Time course of CCI-induced thermal hyperalgesia (Figure 1B) was the same as that of CCI-induced cold hyperalgesia. Value for heat-evoked paw withdrawal latency (PWL) of CCI rats began to decrease on day 4 (7.55 ± 0.90 s vs 7.94 ± 0.69 s, *P *< 0.05), and reached a minimum on day 10 (3.48 ± 0.40 s vs 7.84 ± 0.45 s, *P *< 0.01) post-surgery. Mechanical allodynia was assessed by Von Frey filament assay. Time course of mechanical sensitivity (Figure 1C) following sciatic nerve ligation was also consistent with that of CCI-induced cold sensitivity, except that paw withdrawal threshold (PWT) reached a minimum on day 14 (4.46 ± 1.12 g vs 27.28 ± 1.36 g, *P *< 0.01) following ligature placement. It can be seen that cold and thermal hyperalgesia and mechanical allodynia began on day 4 following CCI and maintained at the peak from day 10 to day 14 after ligature placement.

**Figure 2:**
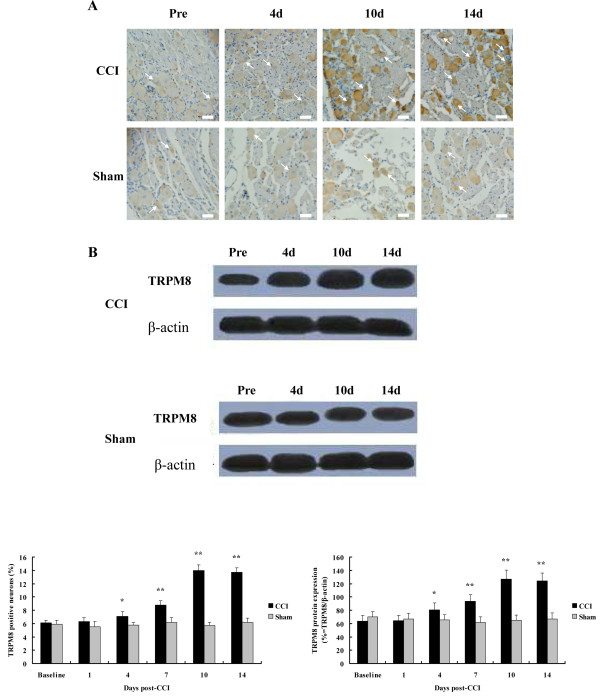
**TRPM8 expression in ipsilateral L5 DRGs of CCI and sham-operated rats**. A, TRPM8 protein staining in L5 DRG sections. Arrows indicate TRPM8 positive neurons. The graph shows the percentage of TRPM8 positive neurons. B, TRPM8- immunoblotting of L5 DRGs. β-actin expression is shown as a control. The graph shows the ratio of density of TRPM8 band to that of β-actin band. Data are expressed as mean ± SEM. *P < 0.05, **P < 0.01 vs. sham-operated rats (t test). Magnification of immunohistochemical images is 400 ×, and scale bars represent 25 μm.

Injury-induced alteration in TRPM8 level in sensory neurons could underlie the development of behavioral sensitivity. Therefore, we examined the alteration of gross expression of TRPM8 protein in ipsilateral L5 DRG before operation (baseline) and on the 1st, 4th, 7th, 10th and 14th day after operation using immunohistochemistry and western blotting. Level of TRPM8 protein was expressed as the percentage of TRPM8 positive neurons relative to total counted DRG neurons in immunohistochemistry or the ratio of density of TRPM8 band to that of β-actin band in western blotting. DRG sections displayed positive staining for TRPM8 mainly in the small-sized neurons, and the distribution of TRPM8 positive neurons was not changed after CCI. Percentages of TRPM8 positive neurons of CCI rats were significantly increased relative to values at corresponding time points of sham-operated rats on day 4 (7.1 ± 0.7%; 52 of 732 neurons; n = 24 fields vs 5.8 ± 0.4%; 42 of 719 neurons; n = 24 fields, *P *< 0.05), day 7 (8.8 ± 0.6%; 71 of 803 neurons; n = 24 fields vs 6.2 ± 0.7%; 49 of 788 neurons; n = 24 fields, *P *< 0.01), day 10 (14.0 ± 0.8%; 110 of 781 neurons; n = 24 fields vs 5.7 ± 0.5%; 46 of 801 neurons; n = 24 fields, *P *< 0.01) and day 14 (13.7 ± 0.7%; 109 of 796 neurons; n = 24 fields vs 6.2 ± 0.6%; 48 of 771 neurons; n = 24 fields, *P *< 0.01) postligature placement (Figure 2A). In immunoblotting, TRPM8 antibody labeled a dense band at approximately 123 kDa, which was consistent with the molecular weight of TRPM8 protein. And β-actin protein, as a control, ran at about 42 kDa. Analytic result of immunoblotting was similar to that of immunohistochemistry. TRPM8 level of CCI rats was first significantly increased on day 4 after surgery (80.52 ± 10.65% vs 65.59 ± 8.10%, *P *< 0.05) compared to that of sham-operated rats, and reached the peak on day 10 (126.78 ± 13.56% vs 64.87 ± 8.01%, *P *< 0.01) and remained significantly elevated on day 14 after operation (123.98 ± 11.73% vs 66.75 ± 9.32%, *P *< 0.01) (Figure 2B).

This part of study revealed that TRPM8 protein expression in L5 DRG ipsilateral to nerve injury was increased from the 4th to 14th day after CCI. This upregulation corresponded well with the development and maintenance of nerve injury-induced hyperalgesia of operated hind paw. These findings imply that the upregulation of TRPM8 protein may be involved in the mechanism of chronic neuropathic pain.

### Effect of menthol on hyperalgesic response and the expression of TRPM8 protein of CCI rats

We have found that the increased expression of TRPM8 protein was related to the hyperalgesia induced by CCI. In order to further elucidate the role of TRPM8 channel in the mechanism of chronic neuropathic pain, we infused menthol, the activator of TRPM8, into the subarachnoid space of CCI rats to investigate the effect of TRPM8 activation on development of hyperalgesia following nerve injury. Menthol or its control, normal saline, was infused into CCI or sham-operated rats through intrathecal catheter on the 14th day after the establishment of pain model, when CCI rats were at peak levels of behavioral sensitization.

**Figure 3:**
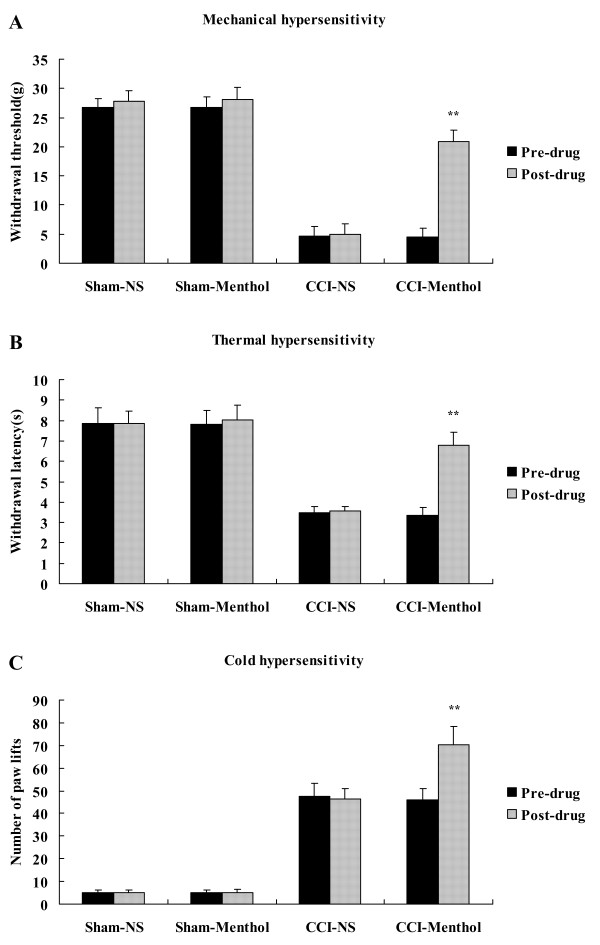
**Effect of menthol on behavioral reflex sensitization of CCI rats**. A, Mechanical sensitivity assessed by Von Frey filament assay. B, Thermal sensitivity assessed by hot plate test. C, Cold sensitivity assessed by cold plate test. Data are expressed as mean ± SEM. **P < 0.01 vs. pre-drug (paired t test).

We observed that application of menthol to CCI rats significantly attenuated mechanical allodynia and thermal hyperalgesia. PWT was increased from 4.54 ± 1.53 g to 20.86 ± 1.90 g (Figure 3A), and PWL from 3.35 ± 0.40 s to 6.79 ± 0.65 s (Figure 3B). But menthol also resulted in an increase of the number of brisk lifts from 46.15 ± 4.98 to 70.37 ± 8.00 (Figure 3C), which implied the enhanced cold sensitivity of CCI rats. Infusion of normal saline to CCI or sham-operated rats, and application of menthol to sham-operated rats all did not produce behavioral changes (Figure 3).

**Figure 4:**
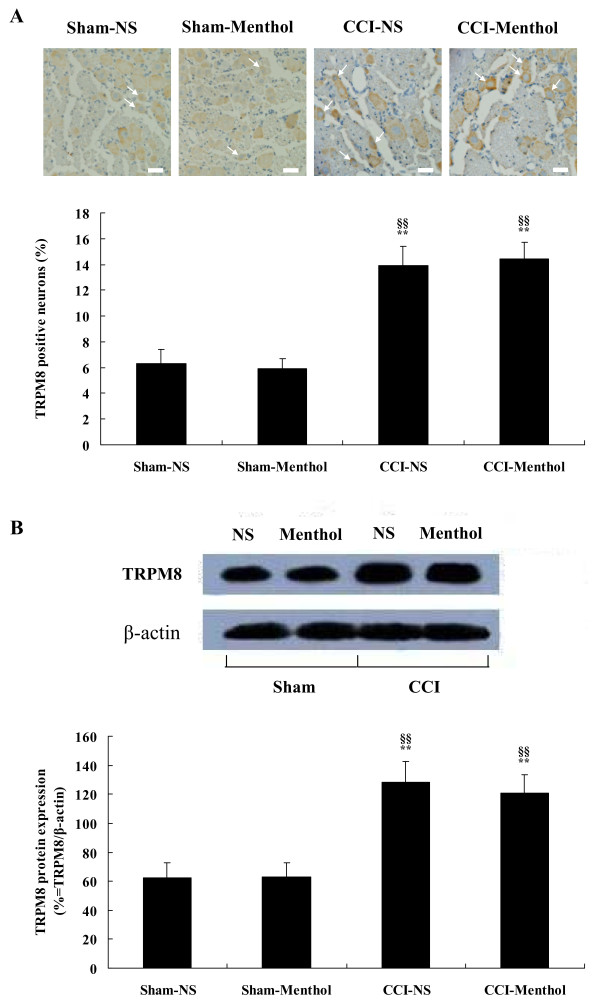
**Effect of menthol on expression of TRPM8 protein in ipsilateral L5 DRGs of CCI rats**. Immunohistochemical staining (A) and western blot analysis (B) of TRPM8 protein in L5 DRGs from sham-operated rats treated with normal saline or menthol and CCI rats treated with normal saline or menthol. Data are expressed as mean ± SEM. **P < 0.01 vs. Sham-NS rats; **^§§^***P *< 0.01 vs. Sham-Menthol rats (one-way ANOVA followed by SNK test). Magnification of immunohistochemical images is 400 ×, and scale bars represent 25 μm. Arrows indicate TRPM8 positive neurons.

Menthol could greatly change the behavioral sensitization of CCI rats, and next, we tried to ascertain whether the influence of menthol on neuropathic reflex sensitization was relevant to the alteration in TRPM8 level. We found that expression levels of TRPM8 protein in CCI rats receiving menthol and CCI rats receiving normal saline were obviously higher than those in sham-operated rats receiving menthol and sham-operated rats receiving normal saline (Figure 4). However, there were no statistical significances in the percentage of TRPM8 positive neurons (Figure 4A) and the ratio of density of TRPM8 band to that of β-actin band (Figure 4B) between menthol-treated CCI rats (14.4 ± 1.3%; 104 of 722; n = 24 fields and 120.65 ± 12.97%, respectively) and normal saline-treated CCI rats (13.9 ± 1.5%; 107 of 769; n = 24 fields and 128.31 ± 14.09%, respectively). This suggested that although intrathecal administration of menthol to CCI rats significantly influenced the hyperalgesic response, it did not affect the total expression of TRPM8 protein in ipsilateral L5 DRG of CCI rats. Percentage of TRPM8 positive neurons did not differ in sham-operated rats infused with menthol or with normal saline (Figure 4), implying that menthol also did not influence the expression of TRPM8 protein in sham-operated rats.

### Effect of TRPM8 gene knockdown on hyperalgesic response of CCI rats

Our study suggested that TRPM8 expression was upregulated after peripheral nerve injury. And we have also investigated the effect of TRPM8 activation on CCI rats. Next, whether selective knockdown of TRPM8 expression affected behavioral reflex sensitization of CCI rats was examined. Here, TRPM8 antisense oligonucleotide (ASODN) was injected intrathecally to knockdown TRPM8 protein, and mismatch oligonucleotide (MMODN) was used as the control of ASODN.

**Figure 5:**
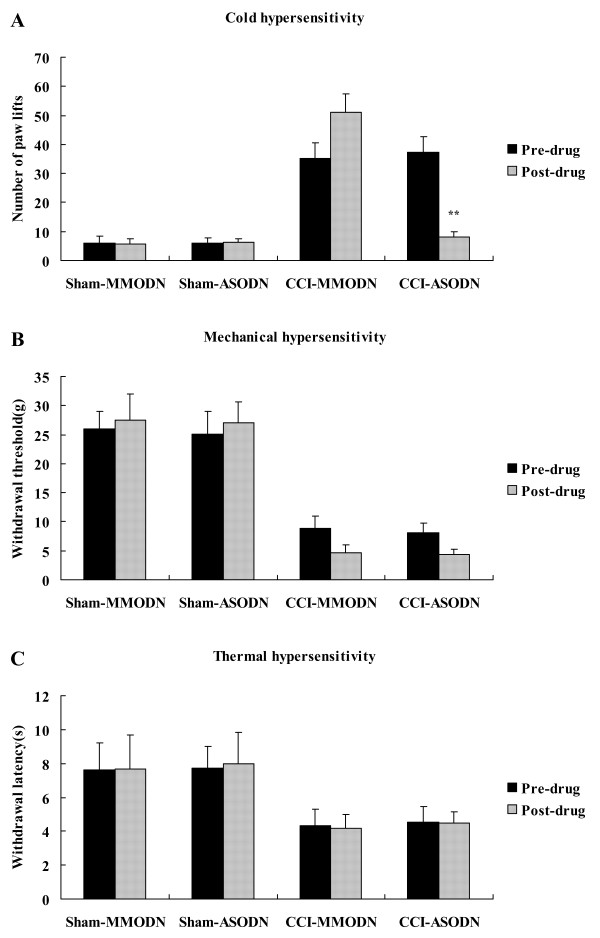
**Effect of TRPM8 ASODN on behavioral reflex sensitization of CCI rats**. A, Cold sensitivity assessed by cold plate test. B, Mechanical sensitivity assessed by Von Frey filament assay. C, Thermal sensitivity assessed by hot plate test. Data are expressed as mean ± SEM. **P < 0.01 vs. CCI-MMODN rats (one-way ANOVA followed by SNK test).

We found that number of brisk lifts of ASODN-treated CCI rats was obviously lower (8.07 ± 1.80 vs 50.99 ± 6.35, *P *< 0.01) than that of MMODN-treated CCI rats (Figure 5A), indicating the application of ASODN alleviated cold hyperalgesia of rats with chronic neuropathic pain. But neither PWT (Figure 5B) nor PWL (Figure 5C) was different between these two groups. There were no statistical differences in value for brisk lifts (Figure 5A), PWT (Figure 5B) and PWL (Figure 5C) between sham-operated rats receiving ASODN and those receiving MMODN.

**Figure 6:**
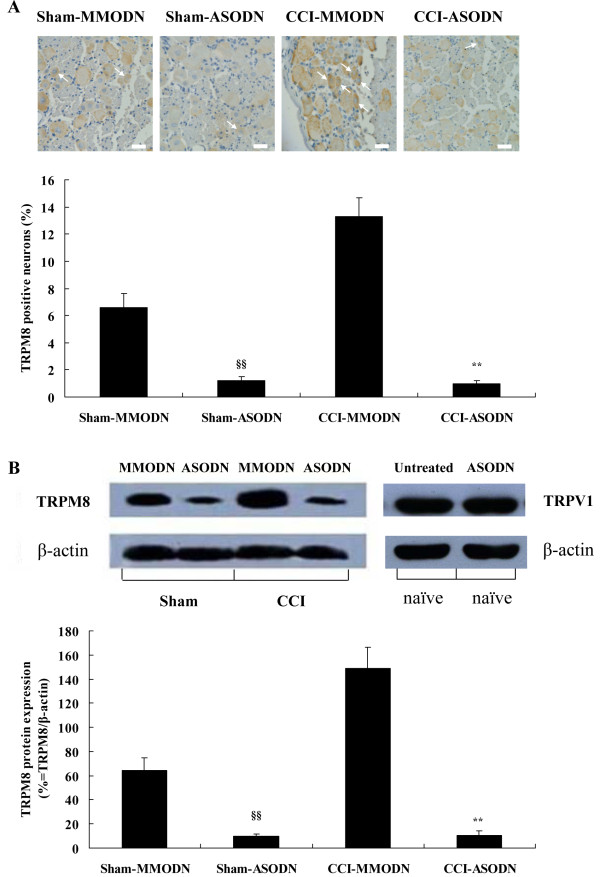
**Effect of TRPM8 ASODN on expression of TRPM8 protein in ipsilateral L5 DRGs of CCI rats**. Immunohistochemical staining (A) and western blot analysis (B) of TRPM8 protein in L5 DRGs from sham-operated rats treated with MMODN or ASODN and CCI rats treated with MMODN or ASODN. TRPV1 immunoreactivity (B) is used to examine the specificity of TRPM8 antisense experiment. Data are expressed as mean ± SEM. **P < 0.01 vs. CCI-MMODN rats; **^§§^***P *< 0.01 vs. Sham-MMODN rats (one-way ANOVA followed by SNK test). Magnification of immunohistochemical images is 400 ×, and scale bars represent 25 μm. Arrows indicate TRPM8 positive neurons.

We showed that the percentage of TRPM8 positive neurons (Figure 6A) and the ratio of density of TRPM8 band to that of β-actin band (Figure 6B) in MMODN-treated CCI rats were 13.3 ± 1.4% (104 of 782 neurons; n = 24 fields) and 148.82 ± 17.38%, respectively. In contrast, values of ASODN-treated CCI rats were significantly lower (1.0 ± 0.2%; 7 of 720 neurons; n = 24 fields and 10.72 ± 3.51%, respectively). Level of TRPM8 protein in sham-operated rats receiving ASODN was also obviously lower than that in sham-operated rats receiving MMODN (Figure 6). Interestingly, there was no statistical significance in the level of TRPM8 protein between ASODN-treated CCI rats (1.0 ± 0.2%; 7 of 720 neurons; n = 24 fields and 10.72 ± 3.51%, respectively) and ASODN-treated sham-operated rats (1.2 ± 0.3%; 9 of 763 neurons; n = 24 fields and 9.56 ± 2.35%, respectively) (Figure 6). In order to examine the specificity of TRPM8 antisense reagent used in our study, we compared the level of TRPV1 protein using immunoblotting in L5 DRG from naïve rats with that from rats intrathecally treated with TRPM8 ASODN during the prior 5 consecutive days. We found that TRPV1 immunoreactivity, at approximately 90 kDa, did not differ between these two kinds of rats, which was informative to confirm the specificity of TRPM8 ASODN in targeting TRPM8 (Figure 6B).

We can see that downregulation of TRPM8 protein induced by ASODN can attenuate cold hyperalgesia of CCI rats, but it had no effect on nerve injury-induced mechanical and thermal hypersensitivity.

## Discussion

The present study investigated the role of cold receptor TRPM8 in primary afferent neurons in the pathogenesis of chronic neuropathic pain. Firstly, we studied the development of sensitized pain behaviors and alteration of TRPM8 expression after CCI to illuminate whether TRPM8 might participate in the mechanism of nerve injury-induced reflex sensitization. Then we observed the effect of TRPM8 activation or down regulation of TRPM8 expression on the nociceptive thresholds of CCI rats by intrathecal administration of menthol or TRPM8 ASODN.

### Upregulation on TRPM8 protein in ipsilateral L5 DRG correlates with pain hypersensitivity following CCI

Immunofluorescence revealed that TRPM8-immunoreactive neurons were significantly increased in ipsilateral L5 DRG of CCI rats [15]. Another study using ribonuclease protection assay showed that CCI induced an increase of TRPM8 RNA in DRG ipsilateral to nerve injury [16]. However, the level of TRPM8 mRNA was decreased in spared nerve injury animals [23]. In our study, we observed the upregulation of TRPM8 protein after CCI. Here, the increase in level of TRPM8 protein was first evident on the 4th day after CCI, and the level reached the peak on the 10th day, and remained significantly elevated on the 14th day postligature placement, concurrent with time course of hyperalgesic response of CCI rats that the decrease of nociceptive thresholds began on day 4 following CCI and persisted up to day 14 post-surgery. It can be seen that the upregulaion of TRPM8 expression in DRG is associated with the mechanism of behavioral sensitization induced by nerve injury.

### Activation of TRPM8 attenuates mechanical and thermal hypersensitivity, but also enhances cold hypersensitivity

Menthol at dose of 100 μg/kg was applied intrathecally in our study, the dose of which was determined according to the result of our preliminary experiment. In one previous study, menthol of 125 μg/kg~260 μg/kg was used to activate TRPM8 receptor [24]. Therefore, menthol of 260 μg/kg was used as the initial dose in our preliminary experiment. We gradually reduced the amount of menthol and found that the hyperalgesic response of CCI rats was also obviously affected even if the dose of menthol was reduced to 90 μg/kg. Because the purpose of our study was to perform qualitative evaluation of the effect of TRPM8 activation on CCI rats, rather than explore the optimal dose of menthol in activating TRPM8, we decided to use menthol of 100 μg/kg in our study in order to facilitate the calculation of the dose of menthol.

Our study demonstrated that intrathecal application of menthol to CCI rats significantly reduced mechanical allodynia and thermal hyperalgesia, which reflected that activation of TRPM8 receptor in the spinal cord inhibited nerve injury-induced behavioral sensitization to mechanical and thermal stimuli. Previous study reported that activation of peripheral TRPM8 receptor induced by topical application of icilin, another TRPM8 agonist, to the paws ipsilateral to nerve injury also produced robust reversal of CCI-induced thermal hyperalgesia and mechanical allodynia [24]. The mechanism by which the activation of TRPM8-mediated signal transduction pathway inhibited neuropathic sensitization may involve Group II and Group III metabotropic Glutamate Receptors (Group II/III mGluRs). Under physiological conditions, synaptic knobs on axons of nociceptive neurons connect with dorsal horn neurons to form synapses, through which the nociceptive information is transmitted. Activation of cool sensory neurons expressing TRPM8 produces synaptic release of glutamate. Then glutamate acts through inhibitory Group II/III mGluRs located either presynaptically on nociceptive neurons or postsynaptically on dorsal-horn neurons, and finally inhibits the input of nociceptive information [24].

In Colburn's study, homozygous TRPM8-deficient (TRPM8^-/-^) mice were generated using the method of homologous recombination, whose physiological properties such as span and behavioral characteristics were not different from those of wild type (TRPM8^+/+^) mice. TRPM8 mRNA and protein levels could not be detected in sensory neurons of TRPM8^-/- ^mice. Prior to CCI, application of acetone to hindpaw of TRPM8^+/+ ^mice caused paw lifting, but acetone could not result in behavioral changes in TRPM8^-/- ^mice. Following sciatic ligation, TRPM8^+/+ ^mice exhibited an enhanced sensitivity to acetone. However, acetone sensitivity of TRPM8^-/- ^mice was not increased at any time after CCI [20]. Besides, Caspani et al [22] observed the activation of TRPM8 by topical application of menthol to the ipsilateral hind paw of CCI mice resulted in the enhancement of hypersensitivity to cold. In our study, cold sensitivity of CCI rats was significantly enhanced after intrathecal administration of menthol. These two studies and ours draw a similar conclusion that TRPM8 may play a crucial role in mediating cold-induced pain after nerve injury. However, Caspani et al [22] also reported that the expression of TRPM8 mRNA in DRG of CCI mice was significantly downregulated. Thus they pointed out that cold hypersensitivity in neuropathic pain states was not dependent on increased expression of TRPM8 in DRG. In contrast, in our experiments, after CCI and sham-operated rats were both intrathecally treated with menthol, cold sensitivity of CCI rats was obviously higher than that of sham-operated rats, and the level of TRPM8 protein in ipsilateral L5 DRG of CCI rats receiving menthol was also significantly higher than that of sham-operated rats receiving menthol, which implies that enhanced cold hypersensitivity after chronic nerve injury may rely on the increased expression of TRPM8 protein in DRG. We consider that the discrepancies in usage method of menthol, topical in theirs and intrathecal in ours, and the research level of molecule, mRNA in theirs and protein in ours, may contribute to the different conclusions about the role of TRPM8 in the formation of neuropathic cold hyperalgesia.

The mechanism of menthol-induced increased cold hyperalgesia in rats with chronic neuropathic pain may be that menthol can lower the voltage required for the activation of TRPM8 channel, which is a voltage sensitive channel activated by membrane depolarization, thereby leading to the enhanced cold sensitivity [25].

### Decreased expression of TRPM8 protein inhibits reflex sensitization to cold after CCI

In the present study, we explored the role of TRPM8 in CCI-induced cold hypersensitivity by reducing the expression of TRPM8 protein. Other researchers also investigated the effect of downregulation of TRPM8 expression on cold hyperalgesia of CCI rats. Proudfoot et al [24] found that the development of CCI-induced cold hyperalgesia was unaffected although TRPM8 protein expression was greatly reduced by TRPM8 ASODN. This discovery is opposite to ours. The reason may be the relatively smaller knockdown amplitude of TRPM8 protein in their study (reduced by 74.6%) compared with that in our study (reduced by 92.8%) after intrathecal ASODN. We intrathecally injected TRPM8 antisense reagent into CCI rats twice a day for 5 consecutive days. But in Proudfoot's research, TRPM8 ASODN was insistently delivered intrathecally over 13 days using minipump containing oligonucleotides which was connected to intrathecal catheter. Although the dose of TRPM8 ASODN used for each rat in Proudfoot's study (168 μg for each rat) is very similar to the dose used in our research (180 μg for each rat), the difference in infusion mode may influence the knockout effect of antisense reagent on TRPM8 protein, and finally leads to two different conclusions.

A comparative study of role in the mechanism of nerve injury-induced cold allodynia between TRPM8 and TRPA1 was also carried out [21]. It was demonstrated that TRPA1 mRNA expression was increased in the nearby uninjured L4 DRG from the 1st to 14th day after L5 spinal nerve ligation (SNL), contemporaneous with the development of L5 SNL-induced neuropathic cold hyperalgesia of the ipsilateral hind paw. However, the expressions of TRPM8 mRNA and protein in the L4 DRG were not changed within the 14 days' trial period. Moreover, L5 SNL-induced cold hyperalgesia was inhibited by intrathecal TRPA1, but not TRPM8 ASODN. Therefore, this study proposed that the increased TRPA1 in uninjured L4 DRG neurons was likely to play a substantial role in the mechanism of cold hyperalgesia in neuropathic states. This point of view is inconsistent with ours that knockdown of the TRPM8 gene by ASODN targeting TRPM8 attenuated nerve injury-induced cold hyperalgesia. Discrepancies in neuropathic pain model and the level of TRPM8 expression may be the reasons for this inconsistence. Intrathecal application of TRPM8 ASODN to SNL rats only lowered the level of TRPM8 protein by 25% [21]. The relatively small decrease in TRPM8 expression is not sufficient to significantly reduce TRPM8-mediated exaggerated sensitivity to cold.

A noteworthy question is it seems to be a paradox that intrathecal injection of menthol into CCI rats not only suppressed mechanical allodynia and thermal hyperalgesia but also enhanced cold hyperalgesia. That is to say, the inhibitory effect of TRPM8 activation on mechanical allodynia and thermal hyperalgesia was achieved at the expense of increased cold sensitivity. We must emphasize that the purpose of this study is to investigate the role of TRPM8 channel in the mechanism of chronic neuropathic pain, and explore whether TRPM8 may be a novel potential target gene for therapy of chronic pain. The result of our animal experiment has proved that some treatments targeting TRPM8 can really relieve chronic pain, suggesting that it is worthwhile to further explore the pathogenesis of chronic neuropathic pain in terms of cold receptor. The next step is to seek ways to make full use of TRPM8-mediated analgesia and minimize even abolish cold hyperalgesia induced by the activation of TRPM8 channel, such as exploring the most appropriate degree of TRPM8 activation or reducing TRPM8-mediated enhanced cold sensitivity by intervening with other targets except TRPM8 channel.

## Conclusions

To those who are suffering from chronic pain, cold stimuli of different degree can bring entirely different perceptions. However, in the process of achieving TRPM8-mediated analgesic effect, it is still not very clear that TRPM8 receptor should be activated or inhibited, and this is also the focus of the present study. Our results show that upregulation of TRPM8 protein corresponds well with the induction and maintenance of sensitized pain responses of ipsilateral hind paw caused by nerve injury. Activation of TRPM8 channel reverses the reflex sensitization to mechanical and thermal stimuli, whereas it enhances cold hyperalgesia as well. On the other hand, downregulation of TRPM8 protein results in the inhibition of cold hypersensitivity, but it makes no impact on nerve injury-induced mechanical allodynia and thermal hyperalgesia. We can see that the function of TRPM8 in the mechanism of hyperalgesic response after nerve injury is extremely crucial and complex. Activating TRPM8 alone will not produce satisfactory analgesia, neither will inhibiting this channel alone. So, in future studies, we will attempt to find ways to avoid hypersensitivity to cold stimuli induced by TRPM8 activation while utilizing TRPM8-mediated analgesia, which should lead to a deeper understanding of the role of TRPM8 in pain, and ultimately produce more effective treatments for chronic neuropathic pain.

## Methods

### Animals

Adult male Sprague-Dawley (SD) rats weighing 250-280 g (aged 7-8 weeks) were provided by Experimental Animal Center of Academy of Military Medical Sciences. Rats were housed in groups of three to four under a standard 12-hour light/dark cycle with access to food and water ad libitum for at least 1 week before the beginning of the experiments. Animal testing procedures and general handling complied with the ethical guidelines and standards established by the institutional animal care and use committee of Tianjin medical University and the local experiment ethics committee.

In the experiment exploring the alteration of TRPM8 protein expression following CCI, ninety-six rats were randomly divided into group CCI and group sham operation (n = 48 each). Eight rats in each group were randomly selected to receive behavioral testing at the following six time points: before operation, on the 1st, 4th, 7th, 10th and 14th day after operation. After behavioral testing at each time point, all eight rats of each group were killed, and ipsilateral L5 DRGs were dissected out for determination of TRPM8.

In the experiment exploring the effect of menthol on CCI rats, thirty-two rats were randomly divided into four groups (n = 8 each): group sham-operated rats with normal saline (Sham-NS), group sham-operated rats with menthol (Sham-Menthol), group CCI rats with normal saline (CCI-NS) and group CCI rats with menthol (CCI-Menthol).

In the experiment exploring the effect of ASODN on CCI rats, thirty-two rats were also randomly divided into four groups (n = 8 each): group sham-operated rats with TRPM8 mismatch oligonucleotide (MMODN) 60 μg/kg (Sham-MMODN), group sham-operated rats with TRPM8 antisense oligonucleotide (ASODN) 60 μg/kg (Sham-ASODN), group CCI rats with MMODN 60 μg/kg (CCI-MMODN) and group CCI rats with ASODN 60 μg/kg (CCI-ASODN).

### Intrathecal catheter placement

A polyethylene catheter was implanted in each rat through foramen magnum according to the method described by Yaksh et al [26]. Twenty-four hours later, rats exhibiting neurological deficits were excluded from the experiments while the rest received 2% lidocaine 20 μl through intrathecal catheter. If the rat exhibited paralysis of hind limb, the catheter was sure to be in the subarachnoid space.

### Construction of CCI model

CCI or sham operation was performed 5 days after intrathecal catheter placement. The surgical procedure was based on that described by Bennett and Xie [27]. Rats were anesthetized using 10% chloral hydrate (350 mg/kg intraperitoneally), and the common sciatic nerve of the right hind paw was exposed at the level of the middle of the thigh by blunt dissection through the biceps femoris. Proximal to the sciatic trifurcation, approximate 7 mm of nerve was freed, and 4 tight ligatures of sterilized 2-0 silk thread were placed around the sciatic nerve with about 1 mm spacing. The desired degree of constriction was to slow down blood flow, without arresting it. In sham-operated animals, the same surgical procedure was followed until the sciatic nerve was exposed, but no ligatures were applied. All surgical procedures were performed by the same person under aseptic conditions.

### Intrathecal administration of drugs

1R, 2S, 5R-(-)-menthol (Bio Basic Inc., Canada) at dose of 100 μg/kg was applied intrathecally in a 20 μl volume of 0.9% normal saline-based vehicle on the 14th day after CCI or sham operation. In case of group sham-operated rats with normal saline and CCI rats with normal saline, equal volume 0.9% normal saline was administered in the same manner. TRPM8 antisense oligonucleotides (ASODN; 5'C*T*CGAAGGACATCTTGCCGTG*G* 3') and mismatch oligonucleotides (MMODN; 5'C*T*GGAAGGACTTCATGCCGTG*C*3') were designed and synthesized by Sangon Biotech Co., Ltd., Shanghai, China, where *represented phosphorothioate linkages. Oligonucleotides at dose of 60 μg/kg were dissolved in 0.9% normal saline to a total volume of 20 μl, and were intrathecally injected into CCI or sham-operated rats twice a day at 8: 00AM and 6:00PM for 5 consecutive days from the 9th to 13th day after CCI or sham operation.

### Behavioral testing

#### Cold sensitivity

The cold plate test was used to determine cold sensitivity in all experiments [27]. Each rat was placed on a copper plate cooled with ice (4 ± 2°C) under a transparent plastic cover and was allowed to acclimate for 5 min. The total number of right hind paw withdrawals from the cold surface not related to general movement was quantified over the subsequent 20 min period.

#### Mechanical sensitivity

Mechanical allodynia was assessed according to the method of Chaplan et al [28]. Paw withdrawal threshold (PWT, g) in response to graded mechanical stimulation was measured using up-down method [29] with von Frey filaments (Stoelting, NorthCoast, USA), which provide a calibrated pressure with incremental stiffness against the hairless skin of the hindpaws.

#### Thermal sensitivity

Thermal hyperalgesia was measured using a 58°C hot plate [30], and expressed as paw withdrawal latency (PWL, s) of right hind paw. Three measurements of thermal nociceptive threshold were taken for each rat, at 4-minute intervals, and the mean of three measurements was regarded as PWL. Maximum latency was defined as 15 s, after which time the animals were removed from the hot plate in order to prevent tissue damage.

Behavioral testing was carried out before and after menthol administration on the 14th day post-surgery. In addition, for the experiment exploring the effect of TRPM8 ASODN on CCI rats, behavioral testing was performed on the 8th and 14th day following operation.

### Immunohistochemistry

Four rats in each group were anaesthetized with 10% chloral hydrate, and then perfused through the aorta with 0.9% normal saline followed by fresh 4% paraformaldehyde in phosphate buffer solution (PBS) (pH 7.4) for 10 min for tissue fixation. Then, lumbar DRGs (n = 4 in each group) at the level of L5 ipsilateral to nerve injury were removed rapidly and placed in 4% paraformaldehyde in PBS for 24 h. L5 DRGs were embedded with paraffin and prepared into slices of 4 μm. Paraffin sections were dewaxed and antigen retrieved for 5 min by microwave heating in citrate buffer, and then cooled to room temperature. Sections were incubated in endogenous peroxidase blocker for 10 min and blocked with normal goat serum for 20 min at room temperature. After that, sections were incubated with TRPM8 primary antibody (1:500, Abcam, UK) overnight at 4°C. Proper antibody diluent (Sigma, USA) was substituted as the primary antibody for the negative controls. Then the sections were incubated with horseradish peroxidase (HRP)-conjugate goat anti-rabbit IgG secondary antibody (1:500, Upstate, USA) for 45 min at 37°C. Followed by DAB coloration, hematoxylin counterstain, gradient dehydration, transparent and cover-slipped, sections were observed using microscope (NIKON ECLIPSE-80i, Nikon, Japan) at 400 ×. For each L5 DRG, three sections were selected for quantification of TRPM8 protein level, which was expressed as the percentage of TRPM8 positive neurons relative to total number of neurons, and two optical fields in each section were randomly captured. Only neurons with clearly visible nuclei for which optical density of TRPM8 immunoreactivity surpassed 2.5 times of the mean cytoplasmic density were counted as positive. The measurement of TRPM8 optical density and data analysis was performed using Image-Pro Plus 6.0 software.

In the experiment exploring the alteration of TRPM8 protein expression following CCI, four rats of each group at each time point (before operation and on the 1st, 4th, 7th, 10th and 14th day after operation) were killed after behavioral testing, and L5 DRGs were removed.

In the experiments exploring the effects of menthol and ASODN on CCI rats, the isolation of DRG was performed after behavioral testing on the 14th day following operation.

### Western blotting

Another four rats in each group were anaesthetized with 10% chloral hydrate, and then only perfused with 0.9% normal saline. Iipsilateral L5 DRGs (n = 4 in each group) were dissected out immediately and cryopreserved in liquid nitrogen. L5 DRGs were minced into fragments and homogenized in lysis buffer (50 mmol/L Tris-HCL pH 6.8, 150 mmol/L NaCl, 5 mmol/L EDTA, 0.5% sodium deoxycholate, 0.5% NP-40 and protease inhibitor cocktail) and centrifuged (2000 g, 5 min, 4°C). The protein concentrations were determined using a BCA Protein Assay reagent kit (Pierce, Rockford, IL). Protein (20 μg) was separated by SDS/PAGE, transferred to PVDF membranes and probed with β-actin primary antibody (1:5000, Abcam, UK) at 4°C overnight. Blots were washed and then incubated with goat anti-mouse IgG secondary antibody conjugated to HRP (1:5000, Abcam, UK) for 1 h at room temperature and signals were visualized by ECL (Amersham, Buckinghamshire, UK). Then the membrane was stripped with restore stripping buffer (Pierce) in order to determine TRPM8. The procedure for determination of TRPM8 was identical to that of β-actin except that primary and secondary antibodies were replaced by polyclonal rabbit anti-rat antibody for TRPM8 (1:500, Abcam, UK) and HRP-conjugate goat anti-rabbit IgG secondary antibody (1:500, Upstate, USA), respectively. As to the determination of TRPV1, its procedure was identical to that of TRPM8 except that the primary antibody was replaced by polyclonal rabbit anti-rat antibody for TRPV1 (1:500, Abcam, UK). TRPM8 or TRPV1 densities were normalized against the concentrations of β-actin, and these ratios were used to analyze the density of bands. Quantitative analysis of the band densities was performed using Quantity One Analyzer 4.5 (Bio- Rad Laboratories, CA) in blinded fashion.

Time points for L5 DRGs dissection in western blotting is exactly the same as that in immunohistochemistry.

### Statistical analysis

The statistical program SPSS 17.0 was used. All data were expressed as mean ± S.E.M.. Differences in behavioral sensitivity as well as TRPM8 expression between CCI rats and sham-operated rats were assessed with two independent sample t-test. Menthol-induced behavioral changes were determined by paired t-test. Any other effects of the drugs were analyzed using one way-analysis of variance (ANOVA) followed by Students-Newman-Keuls (SNK) test for multiple comparisons. A P value < 0.05 was considered statistically significant.

## List of abbreviations

TRPM8: transient receptor potential melastatin 8; CCI: chronic constriction injury; TRP: transient receptor potential; DRG: dorsal root ganglion; PWT: paw withdrawal threshold; PWL: paw withdrawal latency; ASODN: antisense oligonucleotide; MMODN: mismatch oligonucleotide; HRP: horseradish peroxidase; mm: millimeter; min: minute; g: gram; s: second; h: hour; mGluR: metabotropic glutamate receptor; SNL: spinal nerve ligation; SDS/PAGE: sodium dodecyl sulfate/polyacrylamid gel electrophoreses; PBS: phosphate buffer solution.

## Competing interests

The authors declare that they have no competing interests.

## Authors' contributions

SL carried out the behavioral tests, immunochemical experiments and western blot analysis, wrote the manuscript. WC performed drugs injection and the dissection of dorsal root ganglia. SL and RYY participated in data analysis, interpreted the data. XKL revised the manuscript. YYH participated in the design and coordination of the study. WGL designed and coordinated the study, revised the manuscript. All authors read and approved the final manuscript.
